# Microfragmented Fat and Biphasic Calcium Phosphates for Alveolar Cleft Repair: Protocol for a Prospective, Nonblinded, First-in-Human Clinical Study

**DOI:** 10.2196/42371

**Published:** 2024-01-15

**Authors:** Diandra Sabrina Natsir Kalla, Salem Alkaabi, Abul Fauzi, Andi Tajrin, Rifaat Nurrahma, Werner E G Müller, Heinz C Schröder, Xiaohong Wang, Tymour Forouzanfar, Marco N Helder, Muhammad Ruslin

**Affiliations:** 1 Department of Oral and Maxillofacial Surgery/Oral Pathology Amsterdam University Medical Centers and Academic Centre for Dentistry Amsterdam Vrije Universiteit Amsterdam, Amsterdam Movement Sciences Amsterdam Netherlands; 2 Department of Biochemistry Faculty of Medicine Hasanuddin University Makassar Indonesia; 3 Department of Oral and Maxillofacial Surgery Fujairah Hospital Ministry of Health Fujairah United Arab Emirates; 4 Department of Oral and Maxillofacial Surgery Faculty of Dentistry Hasanuddin University Makassar Indonesia; 5 Department of Prosthodontics Faculty of Dentistry Hasanuddin University Makassar Indonesia; 6 ERC Advanced Investigator Grant Research Group at the Institute for Physiological Chemistry University Medical Center of the Johannes Gutenberg University Mainz Germany; 7 Department of Oral and Maxillofacial Surgery Leiden University Medical Centre Leiden Netherlands

**Keywords:** microfragmented fat, calcium phosphate, bone regeneration, regenerative medicine, alveolar, bone grafting, bone, graft, alveolar cleft, surgery, surgical, perioperative, mouth, oral surgery, maxillofacial, jaw, oral pathology, oral, dentistry, dental, tooth, teeth, osteo, osteoconductive biphasic calcium phosphate, autograft, operation

## Abstract

**Background:**

Biphasic calcium phosphates (BCP) may serve as off-the-shelf alternatives for iliac crest-derived autologous bone in alveolar cleft reconstructions. To add osteoinductivity to the osteoconductive BCPs to achieve similar regenerative capacity as autologous bone, a locally harvested buccal fat pad will be mechanically fractionated to generate microfragmented fat (MFAT), which has been shown to have high regenerative capacity due to high pericyte and mesenchymal stem cell content and a preserved perivascular niche.

**Objective:**

Our primary objectives will be to assess the feasibility and safety of the BCP-MFAT combination. The secondary objective will be efficacy, which will be evaluated using radiographic imaging and histological and histomorphometric evaluation of biopsies taken 6 months postoperatively, concomitant with dental implant placement.

**Methods:**

Eight patients with alveolar cleft (≥15 years) will be included in this prospective, nonblinded, first-in-human clinical study. MFAT will be prepared intraoperatively from the patient’s own buccal fat pad. Regular blood tests and physical examinations will be conducted, and any adverse events (AEs) or serious EAs (SAEs) will be meticulously recorded. Radiographic imaging will be performed prior to surgery and at regular intervals after reconstruction of the alveolar cleft with the BCP-MFAT combination. Biopsies obtained after 6 months with a trephine drill used to prepare the implantation site will be assessed with histological and histomorphometric analyses after methylmethacrylate embedding and sectioning.

**Results:**

The primary outcome parameter will be safety after 6 months’ follow-up, as monitored closely using possible occurrences of SAEs based on radiographic imaging, blood tests, and physical examinations. For efficacy, radiographic imaging will be used for clinical grading of the bone construct using the Bergland scale. In addition, bone parameters such as bone volume, osteoid volume, graft volume, and number of osteoclasts will be histomorphometrically quantified. Recruitment started in November 2019, and the trial is currently in the follow-up stage. This protocol’s current version is 1.0, dated September 15, 2019.

**Conclusions:**

In this first-in-human study, not only safety but also the histologically and radiographically assessed regenerative potential of the BCP-MFAT combination will be evaluated in an alveolar cleft model. When an SAE occurs, it will be concluded that the BCP-MFAT combination is not yet safe in the current setting. Regarding AEs, if they do not occur at a higher frequency than that in patients treated with standard care (autologous bone) or can be resolved by noninvasive conventional methods (eg, with analgesics or antibiotics), the BCP-MFAT combination will be considered safe. In all other cases, the BCP-MFAT combination will not yet be considered safe.

**Trial Registration:**

Indonesia Clinical Trial Registry INA-EW74C1N; https://tinyurl.com/28tnrr64

**International Registered Report Identifier (IRRID):**

DERR1-10.2196/42371

## Introduction

Alveolar cleft is defined as a bone gap in the primary palate from the nasal sill to the incisive foramen [1]. The defect occurs as a result of disruption of primary palate development between 4 and 12 weeks of gestational age, specifically in the frontonasal prominence [2]. The treatment protocol varies on the basis of the following factors: timing, surgical procedure, and grafting material. Secondary alveolar bone grafting (SABG) is the most preferred and successful method that is usually performed during the mixed dentition period (6-11 years), which allows the provision of support to teeth eruption and facial growth [1]. The iliac crest as a bone graft donor for alveolar cleft reconstruction has gained popularity since it was first introduced by Schmid [3] in 1954, and, in particular, for SABG procedures because it allows harvesting of large amounts of bone for alveolar cleft surgery [4]. Other bone graft sources include the cranium, tibia, and the mandibular symphysis [5]. However, several studies have reported risks of general postoperative complications using autografts, such as pain, prolonged hospital stay, and donor site–specific complications such as scarring, cutaneous nerve injury near the iliac crest, and hematoma after harvesting the cranial bone [6-9]. Therefore, alternative materials are being evaluated for alveolar cleft surgery.

Biphasic calcium phosphate (BCP) is a bioceramic that consists of 2 materials, hydroxyapatite (HA) and β-tricalcium phosphate, mixed in different ratios [10]. It is a biocompatible, easy-to-handle, safe material with a mineral composition comparable to that of human bone tissue [10]. BCP has been mixed in vivo and in vitro with autografts, inducing factors or cells to improve its osteoinductivity [11,12], also in the fields of dentistry and maxillofacial surgery [13-15]. Although calcium phosphate ceramic is not yet considered standard-of-care, it has been used for alveolar cleft reconstruction with satisfactory results [16], reportedly providing support for teeth eruption [17].

Adipose tissue is a source of mesenchymal stem cells, and adipose stem cells (ASCs) can be collected with minimum risk and discomfort from the buccal fat pad (BFP) [18]. The BFP surrounds the buccinators muscle and other superficial muscles such as the masseter, the zygomaticus major, and the zygomaticus minor [19]. Moreover, multiple studies have shown that the cell yield of ASCs per volume is at least 100-500 times that of mesenchymal stem cells in bone marrow aspirates [18,20]. Commonly, ASCs are prepared using enzymatic (collagenase) digestion which, however, is considered “more than minimal manipulation” of the cells by the US Food and Drug Administration and the European Medicines Agency [21]. An alternative method, which also takes considerably less time, involves processing the adipose tissue mechanically into microfragmented fat (MFAT) [22]. MFAT is reported to have similar or even higher secretory activity of regenerative growth factors and cytokines and pericyte content than an enzymatically derived stromal vascular fraction (SVF) [23]. In addition, the MFAT procedure can be applied even in regular hospitals because its harvesting and processing does not require a major invasive surgery, specialized equipment or expensive disposables, or good manufacturing practices–qualified cell culture expansion. Autologous application of MFAT has, among others, been used with success for clinical reconstructions in the maxillofacial area [24].

We hereby describe the protocol of a first-in-human clinical safety trial using BCP mixed with MFAT for alveolar cleft reconstruction. Our hypothesis is that the combination will be a safe, efficient, and effective alternative to conventional autograft since the osteoconductive BCP is supplemented by the regenerative capacity from the MFAT.

## Methods

### Study Design

**Figure 1:**
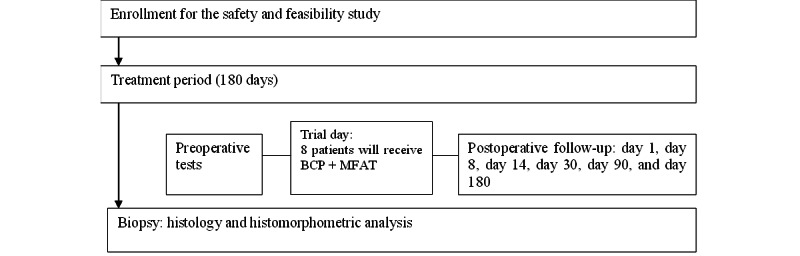
Simplified diagram of the study protocol (adapted from the SPIRIT [Standard Protocol Items: Recommendations for Interventional Trials] checklist; Multimedia Appendix 1). BCP: biphasic calcium phosphate; MFAT: microfragmented fat.

This first-in-human surgical study can be classified as a “stage 1” study in accordance with the IDEAL (innovation, development, exploration, assessment, and long-term study) framework [25]. It is a single-center prospective clinical trial comprising 8 patients, assessing the safety of a combination of MFAT and BCP (BoneCeramic, Straumann) as bone graft material for alveolar cleft reconstruction. The BCP is a synthetic bone graft containing 60% HA and 40% β-tricalcium phosphate, a porosity of 90%, and an interconnected pore size of 100-500 µm. The BCP will be combined in a 1 g:1 cm^3^ ratio with MFAT prepared from the patients’ own BFP, which is processed with a 1.2-mm single-use sizing transfer Tulip Gen II Nanofat Kit (Tulip Medical). The primary end point will be set at 6 months. At each follow-up visit, adverse events (AEs) or serious AEs (SAEs) will be documented, and clinical assessments will be performed at time points specified in the *Interventions* section. After these 6 months, a bone biopsy sample will be taken using a hollow drill during dental implant preparation and subsequently processed for histological or histomorphometric analysis (Figure 1). Finally, a report on safety and proof of concept with regard to bone formation will be made and published.

### Ethical Considerations

The clinical trial protocol was approved by the ethical committee of Hasanuddin University, Makassar, Indonesia (1063/UN4.6.4.5.31/PP36/2019) and registered in the Indonesian trial registry (INA-EW74C1N). Informed consent will be obtained from candidates or their parents or legal guardians who are willing to join the trial after being fully educated about the trial procedure. This study complies with the principles of the Declaration of Helsinki. In the informed consent form signed by the patients or their legal representatives, consent to publish their data in a deidentified manner is included.

### Inclusion and Exclusion Criteria

Patients will be included on the basis of the following criteria [26]: being healthy male or female participants aged ≥15 years, having unilateral alveolar cleft without any previous history of grafting procedures, being categorized as normal healthy patients for anesthetic risk per the American Society for Anesthesiologists’ criteria, and having a normal blood count.

Patients will be excluded on the basis of the following criteria [26]: having poor oral hygiene with mouth plaques; having systemic disease; having systemic or local infection; having received chemotherapy, radiotherapy, immunosuppressives, or anticoagulants that may interfere with the healing process; having received bone growth–inducing factors, malnutrition, or active influenza; and being pregnant.

### Interventions

Under general anesthesia and infiltration with lidocaine (1%) with epinephrine (1:100,000 dilution), the surgeon will identify the Stensen’s duct with a lacrimal probe and make an incision 2-3 cm below the duct [27].﻿ A dissection penetrating the muscles and the superficial fascia will allow spontaneous herniation of the BFP [27]. This procedure will be carried out bilaterally on both cheeks in order to obtain approximately 3 cm^3^ of fat. After vasoconstrictor infiltration with epinephrine (1:100,000), a full mucoperiosteal flap spanning the first molar to the central incisor is lifted. After exposure of the full alveolar cleft and to separate the nasal layer from the oral mucosa, the tissue was meticulously dissected. Following the reflection of a palatal mucoperiosteal flap from either side of the cleft, the palatal tissues are elevated. The oronasal fistula is repaired cranially by elevating and suturing the nasal mucosa [4], thereby creating a pocket for BCP-MFAT deposition.

In parallel with the defect surgery, the harvested fat will be chopped into small pieces with a scissor and soaked in normal saline for 10-15 minutes. The normal saline then will be drained and the chopped fat will be processed into MFAT using 2 syringes (size 10 cm^3^) connected with the 1.2-mm single-use sizing transfer Tulip Gen II Nanofat Kit in accordance with the manufacturer’s protocol. MFAT will be mixed with BCP (Straumann Bone Ceramic) in a ratio of 1 g:1 cm^3^ until it reaches homogenous consistency. The BCP-MFAT mixture will be placed as a graft material into the alveolar cleft defect. If the defect is large and requires more bone graft, another mixture will be prepared with the same mixing ratio. If necessary, a membrane will be used to cover the grafted defect. Finally, the defect will be closed by suturing the palatal mucoperiosteal flaps using absorbable sutures with 3-0 vicryl sutures for mucosa and 4-0 vicryl sutures for nasal reconstruction. All patients will be prescribed antibiotics and analgesics postoperatively.

### AE Assessment

Any change in the health of subjects will be documented in their medical history, and required medical care will be provided. Any unexpected physical or laboratory change, symptom, or disease that occurs in a treated patient who has been administered the graft will be documented as an AE. An AE will be graded in accordance with the World Health Organization’s classification [28] as either serious or nonserious based on its intensity. The Clavien-Dindo Classification of Surgical Complications will also be used in case of any incidence [29]. In the case of an SAE, a report will be made to the sponsor within 24 hours and to the ethical committee within 3 days from the date of onset. If the SAE concerns severe toxicity or infection associated with graft products, the trial will be terminated immediately.

### Sample Size

This is a first-in-human phase I clinical trial aimed to obtain insight on the safety and feasibility of the treatment with the BCP-MFAT combination. We assume that no SAEs or AEs will occur, based on our clinical experience with other applications of MFAT and the well-proven safety of BCP. Upon consultation with a statistician, a sample size of 8 individuals is expected to be sufficient for this trial.

### Recruitment

Patients will be recruited from an existing database of the Hasanuddin University Dental Hospital, from general practices of Hasanuddin Dental Hospital and in the area around Makassar. Thereafter, we will determine whether the candidates fulfill the study’s inclusion and exclusion criteria. Thorough assessment and training regarding the safety measurements at the research site at Hasanuddin University Dental Hospital will be performed prior to the trial by the ethical and surgical teams.

Since we did not want to enroll children in a safety study with this novel concept in clinical practice, we chose to only include older adolescent and adult patients, being themselves capable of decision-making. Within Indonesia, this age group is more common due to cultural and religious backgrounds causing abstinence from cleft surgeries.

The trial will be conducted at Hasanuddin Dental Hospital. All participants will be asked to sign an informed consent form after risks and possible complications of the procedure (eg, bleeding, infection, cheek asymmetry, parotid duct injury, possibility of facial nerve branches injury, and—although not likely—nonclosure) were appropriately communicated with the patient. Data will be handled and stored in a coded—that is, deidentified—format, so that data cannot be traced back to the patient without a decoding key, which is stored in a locked place and only accessible to the study’s principal investigator. Implants will be offered free of charge.

### Randomization and Blinding

Since this trial comprises only 1 type of treatment, no randomization or blinding to the treatment is possible.

### Data Collection and Access

The research team will be informed about the rules and their responsibilities. All members of the research team, which will collect the data in accordance with the evaluation table (Table 1), will receive training on how data collection should be performed. The data manager will document the data in a patient-coded manner (ie, each patient will be assigned a study-specific code under which the data will be stored in order to conceal their identity), which will subsequently be handed over to the clinical evaluators and investigators. The primary end point is set at 6 months.

**Table 1:** Patients’ assessments.

	Consent form	Orthopantomography	Cone-beam computed tomography or computed tomography	Physical examination	Complete blood count	Thermometry	Biopsy
Preoperative	✓	✓	✓	✓	✓	✓	
Operative day				✓		✓	
Postoperative day 1		✓		✓	✓	✓	
Postoperative day 8		✓	✓	✓	✓	✓	
Postoperative day 14				✓		✓	
Postoperative day 30				✓	✓	✓	
Postoperative day 90		✓		✓		✓	
Postoperative day 180		✓	✓	✓	✓	✓	✓

### Posttrial Care

After the primary end point assessment, the participants will be followed up for an additional period of 3 years to ensure their safety and to record whether any delayed side effect occurs as a result of treatment with the BCP-MFAT combination, as previously done in a similar study [26].

### Monitoring

Internal monitors of the Ethics and Research Committee, Faculty of Medicine, Hasanuddin University, will evaluate whether the data are accurately collected. Since negligible risk to the patient is expected as both materials (MFAT and BCP) have been tested in other clinical trials [16,17,24], no data safety monitoring board will be installed. A safety report will be submitted every year to the Medical Research Ethics Committee, Faculty of Medicine, Hasanuddin University. No interim analysis is deemed necessary.

### Amendments

If deemed necessary, amendments to this protocol will be submitted to the ethical committee and competent authority and should be approved prior to implementation to ensure the safety and integrity of participants as well as the scientific value of the trial.

### Evaluation Methods

#### Safety Assessment Based on Physical Examination and Laboratory Measurements

When an SAE occurs, it will be concluded that a combination of MFAT and BCP is not yet safe in the current setting. For AEs, if they do not occur at a higher frequency than that in patients treated with standard care (autologous bone) or can be resolved through noninvasive conventional methods (eg, analgesics or antibiotics), the combination of MFAT and BCP will be considered safe. In all other cases, combination of MFAT and BCP will not be considered safe yet.

#### Radiographic Analysis

To evaluate the success rate of the bone graft, the Bergland scale will be used [30]. This scale will evaluate the integrity and height of the alveolar bone graft and will classify bone height into 4 grades: grade I, bone height is almost normal; grade II, a bone height that is at least 75% of the interalveolar septum; grade III, a bone height of less than 75%; and grade IV, no evidence of bone integration [31].

#### Histological and Histomorphometric Analysis

Histological and histomorphometric analysis will be performed for at least 3 patients who received dental implants after alveolar cleft reconstruction, in accordance with previously published procedures [32]. Briefly, the implant preparation site will be developed using a trephine burr (⌀ 2.0 mm × 10.0 mm in length) that allows biopsy specimen collection from the implant site without interfering with the regular procedure. The biopsy specimens will be fixed in ﻿4% phosphate-buffered formaldehyde, dehydrated in an ascending series of ethanol, and embedded in 80% methylmethacrylate (BDH Chemicals) supplemented with 20% dibuthylphtalate (Merck), 8 g/L lucidol CH-50 L (Akzo Nobel), and 22 μL/10 mL N,N-dimethyl-p-toluidine (Merck). The biopsy specimens will be cut into ﻿5-μm-thick sections and subjected to 2 different staining procedures (﻿Goldner’s trichrome and Tartrate-resistant acid phosphatase staining). Several histomorphometric parameters (bone volume, osteoid volume, graft volume, and number of osteoclasts) will also be measured for quantitative analysis [32]. Two trained examiners will perform the histologic and histomorphometric analyses. In case of dispute, the biopsies will be reanalyzed to reach consensus.

### Statistical Analysis

Since this is a single-arm safety study, statistical analyses will not be performed.

## Results

The primary outcome parameter will be safety after 6 months’ follow-up, assessed by closely monitoring possible occurrences of AEs or SAEs, radiographic imaging, blood tests, and physical examinations. For efficacy, radiographic imaging will be used for clinical grading of the bone construct using the Bergland scale. In addition, bone parameters such as bone volume, osteoid volume, graft volume, and number of osteoclasts will be histomorphometrically quantified. We expect that the feasibility and safety of the procedure will be apparent, as well as its initial efficacy. Recruitment started in November 2019, and the trial is currently in the follow-up stage. This protocol’s current version is 1.0, dated September 15, 2019.

## Discussion

In recent years, there has been increasing interest in the use of adipose tissue for cleft lip and palate reconstruction [33]. Its applicability mostly relies on the quantity of the tissue, the ease of surgical harvesting, and the type of surgical reconstruction in which the tissue is used, for example, correction of cleft lip volume asymmetry [34,35], improvement of velopharyngeal insufficiency after cleft lip and palate repair [36,37], or as an extra flap in cleft palate repair [38-41]. In this study, we will make use of the BFP for bone reconstruction. The BFP is a specialized adipose tissue rich in vascular supply, which is easy to harvest via the oral cavity during an intraoral surgery with minimal morbidity and discomfort [42].

Until now, there are only few reports on the use of adipose tissue as a regenerative compound for bony cleft reconstruction—a phase I clinical trial conducted by Khojasteh et al [24] and an animal study using ASCs for alveolar cleft repair [43]. Both studies used collagenase digestion of the tissue and culture expansion to obtain ASCs for personalized cleft reconstructions. An alternative is the SVF derived from adipose tissue via collagenase digestion, which requires a shorter time frame and may yield similar stem cell–like quantities, allowing intraoperative applications [44,45]. A previous clinical study by Prins et al [44] showed that addition of SVF in an intraoperative setting to calcium phosphate ceramics had an additive value on bone formation, implying that SVF can provide osteoinductivity when combined with calcium phosphate. However, so far, regulatory issues and relative expensive SVF production procedures prohibit its wide applicability [22,23]. Mechanically processed fat or MFAT has emerged as a rapid processing alternative to SVF and is being considered minimally manipulated and thereby less regulation restricted [22,23].

This is the first in human study evaluating a combination of MFAT and biphasic BCP as a regenerative graft for alveolar cleft reconstruction [46,47]. BCP is a ceramic scaffold with a balanced ratio between the less-soluble HA and the more-soluble TCP that results in mechanical and biological properties to support bone and cartilage tissue production [48]. It is sufficient for bone reconstruction in non–load-bearing applications and already accepted as standard of care for certain maxillofacial reconstructions [49].

Recently, calcium phosphate has been applied for alveolar cleft surgeries as well [16,17]. Patients within that study were treated at ages of 9-10 years, which is within the optimum age range for SABG [1]. However, we did not want to enroll children in a safety study with this novel concept in clinical practice. Therefore, although we realize that surgeries at a later age will (1) not make optimal use of the growth spurt and (2) may result in cases having larger or even critical size defects (which will not heal unless supplemented with grafts), we chose to only include older adolescent and adult patients, who are themselves capable of being involved in decision-making. We will perform this study in Indonesia because unoperated patients in this age group are difficult to find in Europe.

This is primarily a safety study, so the main conclusions of the study will be based on safety parameters, particularly on the occurrence of AEs or SAEs.

## Appendix

Multimedia Appendix 1 SPIRIT (Standard Protocol Items: Recommendations for Interventional Trials) checklist.

## Abbreviations

AE: adverse event
ASC: adipose stem cell
BCP: biphasic calcium phosphate
BFP: buccal fat pad
HA: hydroxyapatite
IDEAL: innovation, development, exploration, assessment, and long-term study
MFAT: microfragmented fat
SABG: secondary alveolar bone grafting
SAE: severe adverse event
SVF: stromal vascular fraction

## Data Availability

The data generated in the course of this study will be presented in the main manuscript reporting on the trial outcomes in a deidentified manner or provided in a multimedia appendix coupled with that manuscript.
